# Gender balance and suitable positive actions to promote gender equality among healthcare professionals in neuro-oncology: The EANO positive action initiative

**DOI:** 10.1093/nop/npad064

**Published:** 2023-10-03

**Authors:** Emilie Le Rhun, Florien Boele, Giuseppe Minniti, Norbert Galldiks, Martin Taphoorn, Karin Piil, Roberta Rudà, Simone P Niclou, Marjolein Geurts, Matthias Preusser, Michael Weller, Susan C Short, Linda Dirven

**Affiliations:** Departments of Neurosurgery, Clinical Neuroscience Center, University Hospital and University of Zurich, Zurich, Switzerland; Department of Neurology, Clinical Neuroscience Center, University Hospital and University of Zurich, Zurich, Switzerland; Leeds Institute of Medical Research at St James’s, St James’s University Hospital, University of Leeds, Leeds, UK; Faculty of Medicine and Health, Leeds Institute of Health Sciences, University of Leeds, Leeds, UK; Department of Radiological Sciences, Oncology, and Anatomical Pathology, Sapienza University of Rome, Rome, Italy; Department of Neurology, Faculty of Medicine, University Hospital Cologne, University of Cologne, Cologne, Germany; Institute of Neuroscience and Medicine (INM-3), Research Center Juelich, Juelich, Germany; Center for Integrated Oncology (CIO), Universities of Aachen, Bonn, Cologne, and Duesseldorf, Cologne, Germany; Department of Neurology, Leiden University Medical Center, Leiden, The Netherlands; Department of Oncology, Centre for Cancer and Organ Diseases, Copenhagen University Hospital, Rigshospitalet, Copenhagen, Denmark; Department of People and Technology, Roskilde University, Roskilde, Denmark; Division of Neuro-Oncology, Department of Neuroscience, University of Turin, Turin, Italy; Department of Cancer Research, Luxembourg Institute of Health, Strassen, Luxembourg; Faculty of Science, Technology and Medicine, University of Luxembourg, Esch-sur-Alzette, Luxembourg; The Brain Tumour Center at the Erasmus MC Cancer Institute, Rotterdam, The Netherlands; Division of Oncology, Department of Medicine 1, Medical University, Vienna, Austria; Department of Neurology, Clinical Neuroscience Center, University Hospital and University of Zurich, Zurich, Switzerland; Leeds Institute of Medical Research, University of Leeds, Leeds, UK; Department of Clinical Oncology, Leeds Teaching Hospitals NHS Trust, Leeds, UK; Department of Neurology, Leiden University Medical Center, Leiden, The Netherlands

**Keywords:** Discrimination, Disparity, Female, Indicator, Male

## Abstract

**Background:**

The proportion of women among healthcare and biomedical research professionals in neuro-oncology is growing. With changes in cultural expectations and work-life balance considerations, more men aspire to nonfull-time jobs, yet, leadership positions remain dominated by men.

**Methods:**

The European Association of Neuro-Oncology (EANO) disparity committee carried out a digital survey to explore gender balance and actions suitable to promote gender equality. The survey was distributed among EANO members in 2021, with responses analyzed descriptively.

**Results:**

In total, 262 participants completed the survey (141 women, 53.8%; median age 43). Respondents were neurosurgeons (68, 26.0%); neurologists (67, 25.6%), medical oncologists (43, 16.4%), or other healthcare or research professionals; 208 participants (79.4%) worked full-time. Positive action to enforce the role of women in neuro-oncology was deemed necessary by 180 participants (68.7%), but only 28 participants (10.7%) agreed that *women only* should be promoted until gender balance is reached. A majority of respondents (162, 61.8%) felt that women with an equivalent CV should be prioritized over men to reach gender balance. If in the future the balance favored women at higher positions, 112 respondents (42.7%) agreed to apply positive action for men. The top indicators considered relevant to measure gender balance were: salary for similar positions (183/228, 80.3%), paid overtime (176/228, 77.2%), number of permanent positions (164/228, 71.9%), protected time for research (161/227, 70.9%), and training opportunities (157/227, 69.2%).

**Conclusions:**

Specific indicators may help to measure and promote gender balance and should be considered for implementation among healthcare professionals in neuro-oncology.

In 2019, the European Association of Neuro-Oncology (EANO) created a disparity committee that aims to support diversity in clinical care and research activities in the field of neuro-oncology. The mission of the committee is to address potential disparity issues, including but not limited to gender disparity. A previous survey among EANO and European Organization on Research and Treatment of Cancer (EORTC) members focused on identifying disparity issues and to identify potential strategies to promote gender equality in the field of neuro-oncology.^1^ The results allowed us to conclude that women may experience more difficulties in acquiring leadership positions, that personal preferences may contribute to an underrepresentation of women in leadership positions, and that gender inequalities extend beyond disparities of access to leadership.

Both men and women are exposed to conditions at work that may affect their physical or psychological health. Additionally, both genders face the challenge of finding a balance between work and private life. There is a trend toward a more global sex-neutral workforce contributing to the economy.^2^

To promote equal opportunities for men and women, gender-based *positive action* measures could be introduced. These could focus on removing existing inequalities that affect women’s opportunities. For a measure to be acceptable as positive action for women in employment and education, it should be based on clear and unambiguous criteria and should address specific career inequalities and help women to conduct their professional life on a more equal footing with men.^3^

Compared with other domains of professional life, gender disparities are probably less dominant in medicine. This includes neuro-oncology, where females constitute a large proportion of the workforce already. While the number of women among healthcare professionals is still growing and more men aspire to nonfull-time jobs, the leadership positions remain mainly dominated by males.^4–6^ In this survey, we aimed to evaluate indicators that might potentially be useful to measure gender balance in neuro-oncology and to inquire which actions could be suitable to promote gender equality among healthcare professionals in the field of neuro-oncology.

## Methods

### Design and Participants

A digital survey using SurveyMonkey was developed by the members of the Disparity Committee of EANO and sent out from 2 September, 2021 until 31 December 2021 to the membership of EANO using the email list of the organization. To increase the response rate, a reminder was sent out 2 weeks later, and presidents of national neuro-oncology organizations throughout Europe were contacted with the request to distribute the survey among their respective membership.

The survey was available in English only and consisted of 3 parts, related to: (i) sociodemographic characteristics, comprising 13 questions; (ii) examples of positive actions that could be suitable to promote gender equality, comprising 19 questions; and (iii) indicators that might potentially be useful to measure gender balance, comprising 13 questions. The complete survey can be found in Supplemental File 1. Participation was anonymous.

### Statistical Analyses

Survey answers were exported directly from SurveyMonkey to *SPSS* software (IBM version 25.0) for analysis. Descriptive statistics were used to report the sociodemographic characteristics of the respondents as well as the responses to the categorical items in the survey. For comparisons between men and women, *χ*^2^ tests were used. A *P-*value < 0.05 was considered statistically significant.

## Results

### Respondents

A total of 302 colleagues opened the survey of which 262 colleagues (87%) answered at least one question related to positive action. Only these 262 respondents were taken into account in the statistical analyses.

Out of the 262 respondents, 141 respondents (53.8%) identified themselves as females. Three participants (1%) did not wish to disclose their gender and another 3 individuals did not answer. The median age was 43 years (range: 25–73), and 95% of the respondents were residents of European countries. The majority of respondents were living with a partner or children or both (208/262, 79.4%). Many respondents were neurosurgeons (68/262, 26.0%), neurologists (67/262, 25.6%), or medical oncologists (43/262, 16.4%), and the majority worked full time (208/262, 79.4%). In about half (137/262, 52.3%) of the respondents the main focus was on patient care, in 32.8% (86/262) on both patient care and research equally, and in 14.5% (38/262) mainly on research. Only a minority (26/262, 9.9%) did not spend time on research activities outside office hours, with 21% (55/262) spending >10 h per week (Table 1).

**Table 1.** Sociodemographic Characteristics For All Respondents, and Males and Females Separately.

Respondent Characteristics	All Respondents (*n* = 262)	Women^*^ (*n* = 141)	Men^*^ (*n* = 115)
Age, years Median (range)	43 (25–73)	42 (25–70)	45 (25–73)
Country of residence, *n* (%) Austria France Germany Italy Netherlands Spain Switzerland United Kingdom Other European Non-European Missing	10 (3.8%) 17 (6.5%) 36 (13.7%) 34 (13.0%) 48 (18.3%) 32 (12.2%) 24 (9.2%) 20 (7.6%) 27 (10.3%) 12 (4.6%) 2 (0.8%)	5 (3.5%) 8 (5.7%) 16 (11.3%) 25 (17.7%) 24 (17.0%) 17 (12.1%) 14 (9.9%) 13 (9.2%) 14 (9.9%) 5 (3.5%) –	5 (4.3%) 8 (7.0%) 20 (17.4%) 8 (7.0%) 24 (20.9%) 15 (13.0%) 7 (6.1%) 7 (6.1%) 12 (10.4%) 7 (6.1%) 2 (1.7%)
Living situation, n (%) Living alone, no partner Partner, but living alone Living together with partner Living with partner and children Living with children Other	33 (12.6%) 16 (6.1%) 72 (27.5%) 129 (49.2%) 7 (2.7%) 5 (1.9%)	25 (17.7%) 14 (9.9%) 36 (25.5%) 57 (40.4%) 5 (3.5%) 4 (2.8%)	6 (5.2%) 2 (1.7%) 34 (29.6%) 71 (61.7%) 2 (1.7%) –
Focus of profession, *n* (%) Patient care Research Both patient care and research equally Missing	137 (52.3%) 38 (14.5%) 86 (32.8%) 1 (0.4%)	78 (55.3%) 23 (16.3%) 40 (28.4%) –	56 (48.7%) 14 (12.2%) 44 (38.3%) 1 (0.9%)
Main profession, *n* (%) Epidemiologist Molecular biologist Medical oncologist Neurologist Neuropsychologist Neuroradiologist Neurosurgeon Nurse specialist Pathologist Physiotherapist Radiation oncologist Researcher (clinical) Researcher (preclinical) Speech disorder specialist Other Missing	1 (0.4%) 6 (2.3%) 43 (16.4%) 67 (25.6%) 3 (1.1%) 2 (0.8%) 68 (26.0%) 1 (0.4%) 3 (1.1%) 1 (0.4%) 33 (12.6%) 6 (2.3%) 9 (3.4%) 1 (0.4%) 17 (6.5%) 1 (0.4%)	1 (0.7%) 3 (2.1%) 23 (16.3%) 40 (28.4%) 2 (1.4%) 2 (1.4%) 27 (19.1%) 1 (0.7%) 2 (1.4%) 1 (0.7%) 20 (14.2%) 2 (1.4%) 6 (4.3%) 1 (0.7%) 10 (7.1%) -	– 3 (2.6%) 20 (17.4%) 25 (21.7%) 1 (0.9%) – 38 (33.0%) – 1 (0.9%) – 13 (11.3%) 4 (3.5%) 3 (2.6%) – 6 (5.2%) 1 (0.9%)
Place of work (multiple answers possible), *n* (%) Public hospital Private hospital University hospital University Other institution	67 (25.6%) 10 (3.8%) 169 (64.5%) 22 (8.4%) 17 (6.5%)	43 (30.5%) 7 (5%) 83 (58.9%) 10 (7.1%) 8 (5.6%)	23 (20.0%) 3 (2.6%) 82 (71.3%) 12 (10.4%) 8 (7.0%)
Working time, *n* (%) 10% (0.5 day) 20% (1 day) 30% (1.5 days) 40% (2 days) 50% (2.5 days) 60% (3 days) 70% (3.5 days) 80% (4 days) 90% (4.5 days) 100% (5 days) Missing	– – 3 (1.1%) 1 (0.4%) 1 (0.4%) 3 (1.1%) 5 (1.9%) 28 (10.7%) 12 (4.6%) 208 (79.4%) 1 (0.4%)	– – – 1 (0.7%) – 2 (1.4%) 4 (2.8%) 21 (14.9%) 6 (4.3%) 106 (75.2%) 1 (0.7%)	– – 2 (1.7%) – 1 (0.9%) 1 (0.9%) 1 (0.9%) 7 (6.1%) 6 (5.2%) 97 (84.3%) –
Hours spent per week on, median (range) Patient care Research activities Educational activities Administrative activities	70 (0–100) 20 (0–100) 10 (0–100) 15 (0–100)	70 (0–100) 16 (0–100) 10 (0–72) 15 (0–100)	60 (0–100) 20 (0–100) 10 (0–100) 20 (0–100)
Hours spent per week on research activities outside office hours, *n* (%) 0 h 1–5 h 5–10 h >10 h	26 (9.9%) 109 (41.6%) 72 (27.5%) 55 (21.0%)	16 (11.3%) 72 (51.1%) 30 (21.3%) 23 (16.3%)	10 (8.7%) 36 (31.3%) 39 (33.9%) 30 (26.1%)

^*^ *Sex was missing for 3 respondents and 3 other respondents did not disclose their sex. These respondents are not included in this analysis.*

There were no differences in sociodemographic characteristics between men and women, except for the living situation. Women lived more often alone (17.7% vs 5.2%) and less often with a partner and children (40.4% vs 61.7%) than men, and a lower proportion of women spent >5 h per week outside of office hours on research activities than men (37.6% vs 60.0%).

### Positive Actions to Promote Gender Equality

The results with respect to positive actions to promote gender equality for women are presented in Table 2. The majority of respondents (180/262, 68.7%) indicated that it was relevant (ie, scored as “quite a bit” or “very much” on a 4-point Likert scale ranging from “not at all” to “very much”) to initiate positive action to enforce the role of women in neuro-oncology. There was no statistically significant difference between women and men in this response. If a gender disbalance was identified, respondents thought this should be preferably addressed immediately (121/262, 46.2%). Only a small proportion of respondents (28/262, 10.7%) agreed that *women only* should be considered for certain roles until the gender balance has been reached. If deemed appropriate, this would concern the roles of department chairs in 41.6% (109/262), assistant or associate professors in 37.4% (98/262), or senior physicians in 30.5% (80/262). The majority of respondents (172/262, 65.6%) did not agree with the statement that women with an inferior CV compared with male competitors should be given a position to reach gender balance. In the case of an equivalent CV, only 10.3% (27/262) of respondents indicated that they did not agree that the position should be given to women; instead 162/262 (61.8%) of respondents indicated that this action would be appropriate to reach gender balance. Moreover, most respondents did not agree (176/262, 67.2%) with the statement that women should be put under pressure to accept a position with a high level of responsibility to reach gender balance. Most respondents (112/262, 42.7%) did not agree that higher positions with responsibilities should be fulfilled only by people who work full-time. Respondents were not in agreement on whether there is a risk of limiting career opportunities for women when developing gender equality rules, with 29.8% (78/262) not agreeing compared to 11.5% (30/262) and 8.0% (21/262) agreeing “quite a bit” to “very much,” respectively. Similarly, if the future balance favored women in higher positions, only 42.7% (112/262) of respondents agreed to apply positive action for men, with more women agreeing “quite a bit” or “very much” (51.1% of women vs 20.2% for men, *P* = .014).

**Table 2.** Positive Actions to Promote Gender Equality for Women

Question and answers, *n* (%)	All respondents (*n* = 262)	Women^*^ (*n* = 141)	Men^*^ (*n* = 115)
Do you agree with positive action to enforce the role of women in neuro-oncology?			
Not at all	5 (1.9%)	2 (1.4%)	3 (2.6%)
A little	15 (5.7%)	9 (6.4%)	6 (5.2%)
Neutral	61 (23.3%)	25 (17.7%)	34 (29.6%)
Quite a bit	60 (22.9%)	31 (22.0%)	28 (24.3%)
Very much	120 (45.8%)	74 (52.2%)	43 (37.4%)
Missing	1 (0.4%)	–	1 (0.9%)
When a gender balance issue has been identified, do you believe that the gender balance should be addressed^‡^:			
Immediately	121 (46.2%)	68 (48.2%)	50 (43.5%)
Within 1–5 years	77 (29.4%)	48 (34.0%)	28 (24.3%)
Within 6–10 years	9 (3.4%)	6 (4.3%)	2 (1.7%)
No opinion	54 (20.6%)	19 (13.5%)	34 (29.6%)
Missing	1 (0.4%)	-	1 (0.9%)
Do you agree that higher positions with responsibilities should be fulfilled only by people who work full-time?			
Not at all	112 (42.7%)	68 (48.2%)	42 (36.5%)
A little	32 (12.2%)	20 (14.2%)	12 (10.4%)
Neutral	37 (14.1%)	16 (11.3%)	19 (16.5%)
Quite a bit	46 (17.6%)	22 (15.6%)	23 (20.0%)
Very much	34 (13.0%)	15 (10.6%)	18 (15.7%)
Missing	1 (0.4%)	-	1 (0.9%)
Do you agree that *women only* should be considered for certain roles until the optimal gender balance has been reached?			
Not at all	146 (55.7%)	69 (48.9%)	73 (63.5%)
A little	3 (11.5%)	20 (14.2%)	10 (8.7%)
Neutral	58 (22.1%)	34 (24.1%)	22 (19.1%)
Quite a bit	18 (6.9%)	12 (8.5%)	6 (5.2%)
Very much	10 (3.8%)	6 (4.3%)	4 (3.5%)
Which roles would this be appropriate for?			
Department chair	109 (41.6%)	65 (46.1%)	41 (35.7%)
Assistant or associate professor	98 (37.4%)	56 (39.7%)	40 (34.8%)
Senior physician	80 (30.5%)	41 (29.1%)	37 (32.2%)
Medical student	27 (10.3%)	10 (7.1%)	15 (13.0%)
Do you agree that women with an inferior CV quality for a specific position should be given a position when gender balance needs to be reached?			
Not at all	172 (65.6%)	91 (64.5%)	79 (68.7%)
A little	41 (15.6%)	21 (14.9%)	17 (14.8%)
Neutral	37 (14.1%)	22 (15.6%)	14 (12.2%)
Quite a bit	8 (3.1%)	4 (2.8%)	4 (3.5%)
Very much	3 (1.1%)	2 (1.4%)	1 (0.9%)
Missing	1 (0.4%)	1 (0.7%)	-
Do you agree that women with an equivalent CV quality for a specific position should be given a position when gender balance needs to be reached?			
Not at all	27 (10.3%)	11 (7.8%)	16 (13.9%)
A little	31 (11.8%)	20 (14.2%)	9 (7.8%)
Neutral	40 (15.3%)	19 (13.5%)	19 (16.5%)
Quite a bit	75 (28.6%)	39 (27.7%)	35 (30.4%)
Very much	87 (33.2%)	52 (36.9%)	34 (29.6%)
Missing	2 (0.8%)	-	2 (1.7%)
Do you agree that women should be put under pressure to accept a position with a high level of responsibility if gender balance needs to be reached?			
Not at all	176 (67.2%)	91 (64.5%)	82 (71.3%)
A little	21 (8.0%)	14 (9.9%)	5 (4.3%)
Neutral	32 (12.2%)	16 (11.3%)	16 (13.9%)
Quite a bit	19 (7.3%)	11 (7.8%)	8 (7.0%)
Very much	10 (3.8%)	7 (5.0%)	2 (1.7%)
Missing	4 (4.5%)	2 (1.4%)	2 (1.7%)
Do you agree that there is a risk of limiting career opportunities for women by developing special gender equality rules?			
Not at all	78 (29.8%)	46 (32.6%)	31 (27.0%)
A little	53 (20.2%)	26 (18.4%)	26 (22.6%)
Neutral	75 (28.6%)	43 (30.5%)	30 (20.6%)
Quite a bit	30 (11.5%)	13 (9.2%)	17 (14.8%)
Very much	21 (8.0%)	10 (7.1%)	9 (7.8%)
Missing	5 (1.9%)	3 (2.1%)	2 (1.7%)
If in the future the balance would favor women at higher positions, would you agree to apply positive action for men?^†^			
Not at all	37 (14.1%)	13 (9.2%)	22 (19.1%)
A little	38 (14.5%)	17 (12.1%)	20 (17.4%)
Neutral	71 (27.1%)	38 (27.0%)	32 (27.8%)
Quite a bit	66 (25.2%)	45 (31.9%)	20 (17.4%)
Very much	46 (17.6%)	26 (18.4%)	19 (19.5%)
Missing	4 (1.5%)	2 (1.4%)	2 (1.7%)

^*^Sex was missing for 3 respondents and 3 other respondents did not disclose their sex. These respondents are not included in this analysis.

^†^ *P*-value < .05.

^‡^ *P*-value < 0.01 (for the comparison between females and males).

**Figure 1.**
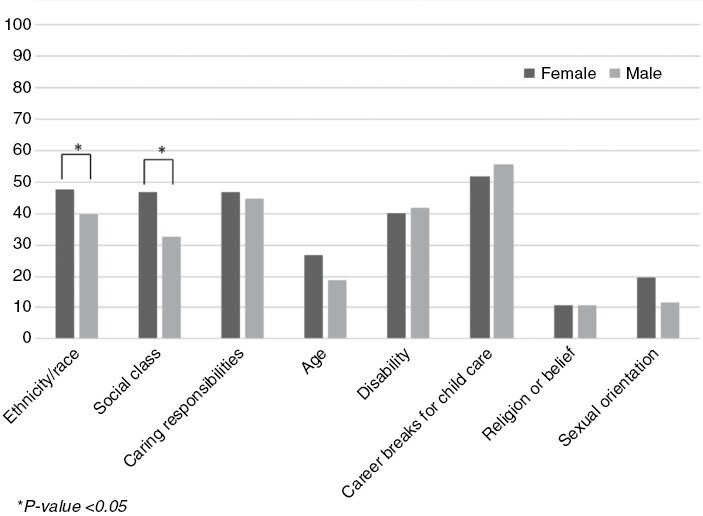
Overview of the percentage of women and men rating the examples of positive actions as relevant (scored as “Quite a bit” or “Very much”).

When asked which topics would be appropriate for positive action (Figure 1, Table S1), respondents rated *career breaks for pregnancy, maternity, paternity, or adoption leave* (135/260, 51.9%), *caring responsibilities* (44.6%, 116/260), *ethnicity/race* (42.9%, 111/259), and *disability* (39.5%, 102/258) as most relevant. *Ethnicity/race* and *social class* were rated significantly more often as relevant (both *P* = .017) by women than by men. *Religion/belief* and *sexual orientation* were rated as least relevant, by 10.4% (27/260) and 15.4% (40/260) of respondents respectively. In the free comments, several respondents indicated that people should be hired based on their competency, skills, qualifications, and merit, regardless of sex or ethnicity.

### Possible Indicators to Measure Gender Balance

**Figure 2.**
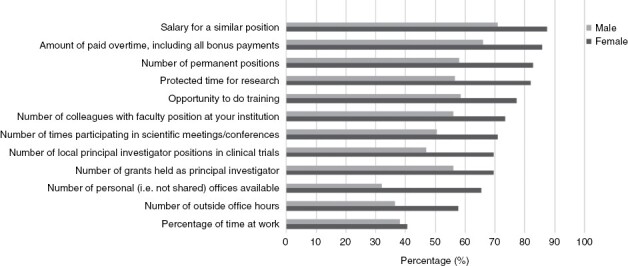
Overview of the percentage of women and men rating the specific indicators to measure gender balance as relevant (scored as “Quite a bit” or “Very much”).

The top five indicators that were deemed relevant (scored as “quite a bit” or “very much”) to measure gender balance in neuro-oncology were *salary for a similar position* (183/228, 80.3%), *amount of paid overtime* (176/228, 77.2%), *number of permanent positions* (164/228, 71.9%), *protected time for research* (161/227, 70.9%) and *opportunity to do training* (157/227, 69.2%). *Number of outside office hours* (110/227, 48.5%) and *percentage of time at work* (89/225, 39.6%) were deemed least relevant. In general, a higher percentage of women rated the possible indicators as relevant compared to men, but the top five indicators were similar (Figure 2). Lastly, 164 of 232 respondents (71%) agreed that the same criteria to measure gender balance should be applied to men in the situation where women outnumber men in higher positions (Table 3).

**Table 3.** Possible Indicators to Measure the Gender Balance in Neuro-Oncology

Question, *n* (%)	All Respondents (*n* = 262)	Women^*^ (*n* = 141)	Men^*^ (*n* = 115)
In your opinion, should the following aspects be used to measure gender balance (ie, measured separately for women and men, and subsequently be compared)?			
Percentage of time at work?			
Not at all	61 (23.3%)	32 (22.7%)	27 (23.5%)
A little	27 (10.3%)	18 (12.8%)	8 (7.0%)
Neutral	51 (19.5.%)	26 (18.4%)	25 (21.7%)
Quite a bit	51 (19.5%)	29 (20.6%)	20 (17.4%)
Very much	40 (15.3%)	23 (16.3%)	17 (14.8%)
Missing	32 (12.2%)	13 (9.2%)	18 (15.7%)
Salary for a similar position?^†^			
Not at all	17 (6.5%)	6 (4.3%)	10 (8.7%)
A little	6 (2.3%)	2 (1.4%)	4 (3.5%)
Neutral	23 (8.8%)	8 (5.7%)	15 (13.0%)
Quite a bit	41 (15.6%)	23 (16.3%)	16 (13.9%)
Very much	146 (55.7%)	89 (63.1%)	55 (47.8%)
Missing	29 (11.1%)	13 (9.2%)	15 (13.0%)
Amount of paid overtime, including all bonus payments?^†^			
Not at all	21 (8.0%)	8 (5.7%)	13 (11.3%)
A little	5 (1.9%)	1 (0.7%)	3 (2.6%)
Neutral	28 (10.7%)	9 (6.4%)	18 (15.7%)
Quite a bit	53 (20.2%)	33 (23.4%)	18 (15.7%)
Very much	126 (48.1%)	77 (54.6%)	48 (41.7%)
Missing	29 (11.1%)	13 (9.2%)	15 (13.0%)
Number of permanent positions?^‡^			
Not at all	20 (7.6%)	3 (2.1%)	17 (14.8%)
A little	6 (2.3%)	2 (1.4%)	3 (2.6%)
Neutral	40 (15.3%)	17 (12.1%)	22 (19.1%)
Quite a bit	63 (24.0%)	37 (26.2%)	23 (20.0%)
Very much	104 (39.7%)	69 (48.9%)	35 (30.4%)
Missing	29 (11.1%)	13 (9.2%)	15 (13.0%)
Number of colleagues with faculty positions at your institution?^†^			
Not at all	24 (9.2%)	7 (5.0%)	17 (14.8%)
A little	8 (3.1%)	5 (3.5%)	2 (1.7%)
Neutral	48 (18.3%)	22 (15.6%)	25 (21.7%)
Quite a bit	61 (23.3%)	36 (25.5%)	22 (19.1%)
Very much	92 (35.1%)	58 (41.1%)	34 (29.6%)
Missing	29 (11.1%)	13 (9.2%)	15 (13.0%)
Number of outside office hours?^†^			
Not at all	47 (17.9%)	20 (14.2%)	27 (23.5%)
A little	17 (6.5%)	8 (5.7%)	8 (7.0%)
Neutral	55 (21.0%)	26 (18.4%)	28 (24.3%)
Quite a bit	51 (19.5%)	30 (21.3%)	19 (16.5%)
Very much	62 (23.7%)	44 (31.2%)	17 (14.8%)
Missing	30 (11.5%)	13 (9.2%)	16 (13.9%)
Number of personal (ie, not shared) offices available?^†^			
Not at all	47 (17.9%)	19 (13.5%)	27 (23.5%)
A little	19 (7.3%)	12 (8.5%)	7 (6.1%)
Neutral	69 (26.3%)	33 (23.4%)	34 (29.6%)
Quite a bit	45 (17.2%)	28 (19.9%)	15 (13.0%)
Very much	52 (19.8%)	35 (24.8%)	17 (14.8%)
Missing	30 (11.5%)	14 (9.9%)	15 (13.0%)
Number of grants held as a principal investigator?^‡^			
Not at all	22 (8.4%)	5 (3.5%)	17 (14.8%)
A little	16 (6.1%)	11 (7.8%)	4 (3.5%)
Neutral	46 (17.6%)	23 (16.3%)	23 (20.0%)
Quite a bit	80 (30.5%)	46 (32.6%)	31 (27.0%)
Very much	69 (26.3%)	43 (30.5%)	25 (21.7%)
Missing	29 (11.1%)	13 (9.2%)	15 (13.0%)
Number of local principal investigator positions in clinical trials?^‡^			
Not at all	22 (8.4%)	5 (3.5%)	16 (13.9%)
A little	20 (7.6%)	11 (7.8%)	9 (7.8%)
Neutral	51 (19.5%)	23 (16.3%)	28 (24.3%)
Quite a bit	79 (30.2%)	50 (35.5%)	26 (22.6%)
Very much	61 (23.3%)	39 (27.7%)	21 (18.3%)
Missing	29 (11.1%)	13 (9.2%)	15 (13.0%)
Number of times participating in scientific meetings/ conferences supported by the institution?^†^			
Not at all	22 (8.4%)	6 (4.3%)	16 (13.9%)
A little	24 (9.2%)	11 (7.8%)	13 (11.3%)
Neutral	41 (15.6%)	20 (14.2%)	20 (17.4%)
Quite a bit	78 (29.8%)	48 (34.0%)	27 (23.5%)
Very much	67 (25.6%)	43 (30.5%)	23 (20.0%)
Missing	30 (11.5%)	13 (9.2%)	16 (13.9%)
Opportunity to do training?^‡^			
Not at all	17 (6.5%)	2 (1.4%)	15 (13.0%)
A little	14 (5.3%)	10 (7.1%)	4 (3.5%)
Neutral	40 (15.3%)	17 (12.1%)	22 (19.1%)
Quite a bit	77 (29.4%)	49 (34.8%)	25 (21.7%)
Very much	84 (32.1%)	50 (35.5%)	33 (28.7%)
Missing	30 (11.5%)	13 (9.2%)	16 (13.9%)
Protected time for research?^‡^			
Not at all	19 (7.3%)	3 (2.1%)	16 (13.9%)
A little	12 (4.6%)	9 (6.4%)	3 (2.6%)
Neutral	36 (13.7%)	11 (7.8%)	24 (20.9%)
Quite a bit	69 (26.3%)	43 (30.5%)	24 (20.9%)
Very much	96 (36.6%)	62 (44.0%)	32 (27.8%)
Missing	30 (11.5%)	13 (9.2%)	16 (13.9%)
Lastly, would you accept the same criteria to maintain gender balance for men when women will outnumber men in higher positions?			
Not at all	19 (7.3%)	5 (3.5%)	13 (11.3%)
A little	13 (5.0%)	8 (5.7%)	5 (4.3%)
Neutral	36 (13.7%)	16 (11.3%)	18 (15.7%)
Quite a bit	51 (19.5%)	29 (20.6%)	21 (18.3%)
Very much	113 (43.1%)	70 (49.6%)	42 (36.5%)
Missing	30 (11.5%)	13 (9.2%)	16 (13.9%)

^*^Sex was missing for 3 respondents and 3 other respondents did not disclose their sex. These respondents are not included in this analysis.

^†^ *P*-value < .05.

^‡^ *P*-value < .01 (for the comparison between females and males).

## Discussion

The present study is part of the efforts of EANO to understand the role of gender imbalances and their impact on the development of a balanced, qualified workforce serving patients, caregivers, and research in neuro-oncology across Europe. Given the membership of EANO of 659 individuals in 2021, the response by 262 individuals indicates that this topic is considered to be of interest. The majority of responders came from clinical neuro-oncology, reflecting the membership of EANO. Since the survey was also distributed across national neuro-oncology groups, we have no information on how many respondents were actually EANO members.

The work profile characteristics of respondents indicate that neurosurgeons are more often men (23/43: 53%), whereas respondents were more often women in other disciplines (neurology: 40/65: 62%; radiation oncology: 20/33: 61%, medical oncology: 23/43: 53%). Women live more often alone and allocate less time to research activities outside of working hours than men (Table 1). There was generally a positive view on action to support the career opportunities of women in neuro-oncology (Table 2). However, a majority of responders would not support that women with an inferior CV would be given priority for positions until a gender balance has been achieved. Interestingly, once women might have taken over the majority of higher positions, there was still no majority to apply positive action for men, at least in the current situation where the survey was conducted.

Most of the parameters proposed in the survey to assess whether the gender balance has been achieved were considered useful by the respondents (Table 3), but for some statements, including the same salary for a similar position or the same compensation for paid overtime, one might argue that these should be implemented as indicators of balance immediately, if not already equal between genders. To obtain permanent positions and secure protected time for research may require more time and training and education opportunities for women notably in some countries where the academic workforce is still very dominated by men at present, but these figures should already be monitored.

Limitations to positive discrimination include the need to consider biological differences, such as physical strength, but these should not be overrated.^2^ Pregnancy and parental leave will continue to shape career paths, likely in a profession- and country-specific manner. Personal choices of lifestyle and career choices have also to be considered. Both men and women engaged in neuro-oncology are regularly confronted with severely ill and understandably demanding patients and may be exposed to sources of irradiation and potentially toxic agents when administering tumor pharmacotherapy. As seen in this survey, more than 20% of respondents declared spending more than 10 hours on research activities outside of office hours, demonstrating their personal engagement. Looking at the results directly, most respondents did not feel that full-time employment was required to assume leadership roles.

Surveys such as the present one have inherent limitations. The sample size was rather small, given the likely heterogeneity of age, profession, country, and setting of work positions. We did not learn about the attitudes of the large number of colleagues who were invited but decided not to respond. Further, surveys collect opinions and experiences which are necessarily subjective, and not data. Importantly, the survey did not address an association between gender and quality of care. It has been claimed that the rates of morbidity and mortality are lower with female than with male physicians in certain domains of medicine^7–9^ but such analyses need to be interpreted with caution, and it remains open whether considerations apply to contemporary neuro-oncology.

The current recommendation of some institutions, such as the EU Clinical Trials Expert Group (https://ec.europa.eu/transparency/expert-groups-register/screen/home?lang=en), to use curriculum vitae without personal data, including omission of birth date, gender, nationality, would limit positive discriminatory actions. Lastly, positive discrimination and follow-up of key indexes, even if encouraged by professional societies, can currently probably only be performed at the institutional level.

In light of this, EANO can contribute significantly by prioritizing the promotion of training and mentoring for motivated females. Concentrating efforts in this area would be a reasonable task to undertake. Gender balance serves as a powerful source of inspiration and motivation for future generations of healthcare professionals. It helps create an environment where individuals are evaluated based on their skills, qualifications, and expertise rather than predetermined gender roles. The disparity committee of EANO takes a proactive stance in fostering gender balance, equity, and inclusivity within various programs such as mentoring and webinar series. This vision strengthens the field and creates opportunities for individuals to excel based on their abilities, ultimately benefiting, and advancing the field of neuro-oncology as a whole.

## Supplementary Material

npad064_suppl_Supplementary_Tables_S1
