# Supplementary material for: Gender balance and suitable positive actions to promote gender equality among healthcare professionals in neuro-oncology: The EANO positive action initiative
Source: Neurooncol Pract. 2023 Oct 3;11(1):46–55. doi: 10.1093/nop/npad064 (PMC10785600; doi:10.1093/nop/npad064)
Supplement: npad064_suppl_Supplementary_Tables_S1 [file npad064_suppl_supplementary_tables_s1.docx]

**Table S1. Relevance of possible topics for positive action to address gender inequality in neuro-oncology.**

| **Question, n (%)** | **All respondents**  **(n=262)** | **Women***  **(n=141)** | **Men***  **(n=115)** | |
| --- | --- | --- | --- | --- |
| Do you think that the following potential sources of inequality should also be taken into account at work (as a topic for positive action)? | | | |  |
| *Ethnicity / race?* |  |  |  |  |
| Not at all  A little  Neutral  Quite a bit  Very much  Missing | 75 (28.6%)  33 (12.6%)  38 (14.5%)  58 (21.8%)  56 (21.4%)  3 (1.1%) | 31 (22.0%)  21 (14.9%)  21 (14.9%)  39 (27.7%)  27 (19.1%)  2 (1.4%) | 42 (36.5%)  11 (9.6%)  16 (13.9%)  17 (14.8%)  28 (24.3%)  1 (0.9%) | |
| *Social class?^†^* |  |  |  | |
| Not at all  A little  Neutral  Quite a bit  Very much  Missing | 86 (32.8%)  29 (11.1%)  53 (20.2%)  47 (17.9%)  44 (16.8%)  3 (1.1%) | 36 (25.5%)  16 (11.3%)  34 (24.1%)  32 (22.7%)  21 (14.9%)  2 (1.4%) | 48 (41.7%)  12 (10.4%)  17 (14.8%)  15 (13.0%)  22 (19.1%)  1 (0.9%) | |
| *Caring responsibilities?^†^*  Not at all  A little  Neutral  Quite a bit  Very much  Missing | 46 (17.6%)  32 (12.2%)  64 (24.4%)  66 (25.2%)  51 (19.5%)  3 (1.1%) | 18 (12.8%)  18 (12.8%)  38 (27.0%)  39 (27.7%)  26 (18.4%)  2 (1.4%) | 28 (24.3%)  13 (11.3%)  22 (19.1%)  27 (23.5%)  24 (20.9%)  1 (0.9%) | |
| *Age?*  Not at all  A little  Neutral  Quite a bit  Very much  Missing | 73 (27.9%)  57 (21.8%)  69 (26.3%)  42 (16.0%)  16 (6.1%)  5 (1.9%) | 35 (24.8%)  28 (19.9%)  39 (27.7%)  28 (19.9%)  9 (6.4%)  2 (1.4%) | 35 (30.4%)  28 (24.3%)  28 (24.3%)  14 (12.2%)  7 (6.1%)  3 (2.6%) | |
| *Disability?*  Not at all  A little  Neutral  Quite a bit  Very much  Missing | 52 (19.8%)  30 (11.5%)  71 (27.1%)  56 (21.4%)  47 (17.9%)  6 (2.3%) | 27 (19.1%)  15 (10.6%)  40 (28.4%)  29 (20.6%)  26 (18.4%)  4 (2.8%) | 25 (21.7%)  14 (12.2%)  27 (23.5%)  27 (23.5%)  20 (17.4%)  2 (1.7%) | |
| *Career breaks due to pregnancy, maternity, paternity or adoption leave?* | | | |  |
| Not at all  A little  Neutral  Quite a bit  Very much  Missing | 40 (15.3%)  40 (15.3%)  42 (16.0%)  74 (28.2%)  62 (23.7%)  4 (1.5%) | 22 (15.6%)  21 (14.9%)  24 (17.0%)  36 (25.5%)  36 (25.5%)  2 (1.4%) | 17 (14.8%)  17 (14.8%)  16 (13.9%)  38 (33.0%)  25 (21.7%)  2 (1.7%) | |
| *Religion or belief?*  Not at all  A little  Neutral  Quite a bit  Very much  Missing | 150 (57.3%)  19 (7.3%)  62 (23.7%)  12 (4.6%)  15 (5.7%)  4 (1.5%) | 72 (51.1%)  12 (8.5%)  40 (28.4%)  8 (5.7%)  7 (5.0%)  2 (1.4%) | 74 (64.3%)  6 (5.2%)  21 (18.3%)  4 (3.5%)  8 (7.0%)  2 (1.7%) | |
| *Sexual orientation?*  Not at all  A little  Neutral  Quite a bit  Very much  Missing | 147 (56.1%)  13 (5.0%)  58 (22.1%)  18 (6.9%)  22 (8.4%)  4 (1.5%) | 69 (48.9%)  7 (5.0%)  36 (25.5%)  13 (9.2%)  14 (9.9%)  2 (1.4%) | 75 (65.2%)  5 (4.3%)  20 (17.4%)  5 (4.3%)  8 (7.0%)  2 (1.7%) | |
| *Other?*  Beauty | 1 (0.4%) | 1 (0.7%) | - | |

**Sex was missing for 3 respondents and 3 other respondents did not disclose their sex. These respondents are not included in this analysis.*
